# Unveiling the Evolutionary Lineages and Habitat Dynamics of the Monotypic Crowned River Turtle *Hardella thurjii* (Gray, 1831) (Testudines: Geoemydidae): Strategic Conservation Insights for an Endangered Freshwater Turtle From Southern Asia

**DOI:** 10.1002/ece3.71530

**Published:** 2025-06-20

**Authors:** Imon Abedin, Arunima Singh, Jayaditya Purakayastha, Shailendra Singh, Kulendra Chandra Das, Hyun‐Woo Kim, Hye‐Eun Kang, Shantanu Kundu

**Affiliations:** ^1^ Dibru‐Saikhowa Conservation Society Tinsukia India; ^2^ Turtle Survival Alliance Foundation India Lucknow India; ^3^ Help Earth Guwahati Assam India; ^4^ Department of Environmental Science Pachhunga University College Aizawl Mizoram India; ^5^ Department of Marine Biology Pukyong National University Busan Republic of Korea; ^6^ Marine Integrated Biomedical Technology Center, National Key Research Institutes in Universities Pukyong National University Busan Republic of Korea; ^7^ Department of Biology, Faculty of Science and Technology Airlangga University Surabaya Indonesia; ^8^ Institute of Marine Life Science Pukyong National University Busan Republic of Korea; ^9^ Ocean and Fisheries Development International Cooperation Institute, College of Fisheries Science Pukyong National University Busan Republic of Korea; ^10^ International Graduate Program of Fisheries Science Pukyong National University Busan Republic of Korea

**Keywords:** chelonians, conservation, mitogenome, phylogeny, species distribution modeling, threatened species

## Abstract

The matrilineal evolutionary history and habitat preferences of the monotopic freshwater turtle *Hardella thurjii* remain largely unexplored, posing challenges for the development of precise and effective conservation strategies. This study provides the first complete mitochondrial genome sequence of *H. thurjii* (16,699 bp), encompassing 13 protein‐coding genes (PCGs), 22 transfer RNAs, two ribosomal RNAs, and an AT‐rich control region (CR). Most PCGs are initiated by ATG, except for cytochrome c oxidase subunit I gene (*COI*), which uses GTG, with eight PCGs having complete termination codons and five exhibiting incomplete stop codons. The CR of *H. thurjii* exhibits a distinctive structural organization, characterized by conserved sequence blocks and three consensus tandem repeats, distinguishing it from other Batagurinae species. The phylogenetic analyses based on Bayesian inference and maximum‐likelihood approaches using PCGs reveal a sister relationship between *H. thurjii* and other *Batagur* species, further corroborating the monophyletic status of the subfamily Batagurinae. Further, species distribution modeling with an ensemble approach effectively maps the global habitat suitability of *H. thurjii* for conservation planning under current and future climates. The model identified 110,490 km^2^ of suitable habitat in the present scenario, with 35,757 km^2^ in the eastern range and 83,723 km^2^ in the western range. Notably, future climate projections indicate a 32.38% overall increase in suitable habitat, primarily in the eastern range, while the western range faces a decline in habitat suitability. This contrasting pattern altered habitat geometry dynamics, increasing the size, number, and connectivity of patches in the eastern range while reducing and fragmenting them in the western range. By integrating mitogenomic and habitat suitability analyses, this study offers valuable insights into the past evolutionary history and current ecological preferences of endangered *H. thurjii* , aiding the development of effective conservation and management strategies for this species and other freshwater turtles globally.

## Graphical abstract

This study provides the first complete mitochondrial genome of the monotypic *H. thurjii* , sequenced using next‐generation technologies. Additionally, it employs ensemble species distribution modeling to evaluate habitat suitability and shape geometry under both current and projected climatic scenarios. This integrated approach offers valuable insights for developing effective conservation strategies for the endangered *H. thurjii* in South Asia.

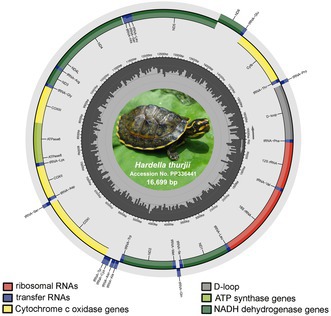

## 1 Introduction

Over the past century, the rate of species extinction has risen sharply, with life on Earth now confronting a sixth mass extinction event driven by human activities, climate change, and ecological collapse (Teixeira and Huber 2021). Consequently, safeguarding biodiversity has become a critical priority to sustain ecosystems and human well‐being, necessitating the adoption of a unified conceptual framework and the implementation of effective conservation strategies (Conde et al. 2019). This situation is driven by a combination of ecological and anthropogenic factors and is further exacerbated by the accelerating pace of global climate change (Pimm et al. 1995; Mothes et al. 2020; Urban 2015). Such hostile conditions in the global hydrological cycle are causing significant alterations in the availability and distribution of inland water resources, directly impacting freshwater ecosystems (Huntington 2006; Barbarossa et al. 2021; Lintermans et al. 2024). For example, the riverine systems originating in the Himalayan region and their associated biodiversity are particularly vulnerable to these environmental pressures in South and Southeast Asia (Uereyen et al. 2022). These riverine systems are critical for supporting diverse biodiversity components that play a pivotal role in maintaining ecosystems essential for human well‐being across the Indian subcontinent (Wijngaard et al. 2018; Biemans et al. 2019).

Concurrently, a rapid global freshwater crisis is unfolding, with many freshwater ecosystems disappearing at an alarming rate (Reid et al. 2019; Sayer et al. 2025). This accelerated degradation is driving the widespread extirpation of biodiversity within these systems (Albert et al. 2021). This degradation of freshwater habitats has had particularly severe impacts on freshwater turtles, among other components of aquatic biodiversity. As some of the oldest living animals, these species are experiencing dramatic population declines, with many now classified among the most threatened species on Earth (Butler 2019; Willey et al. 2022). Thus, protecting these species is crucial, as they play essential roles in aquatic ecosystems by contributing to key ecological processes, including food web dynamics, scavenging activities, etc. (Santori et al. 2020). In addition to habitat loss and degradation, freshwater turtles face significant threats from direct exploitation, including killings for bushmeat, harvesting for the pet trade, and the use of their body parts in traditional medicines in international markets (Gibbons et al. 2000; Stanford et al. 2020). However, conservation efforts for many threatened freshwater turtles remain largely neglected, especially in Asian countries, due to a lack of comprehensive, multi‐dimensional species information (Tilman et al. 2017; Harfoot et al. 2021).

The family Geoemydidae represent one of the most diverse groups of turtles, encompassing three subfamilies, divided into 19 genera and 71 valid species (TTWG Turtle Taxonomy Working Group 2021). These turtles exhibit a broad geographic distribution, spanning Asia, Europe, North Africa, Central and South America, as well as inhabits a wide range of environments, from fully aquatic to predominantly semi‐aquatic habitats (Iverson 1992). Unfortunately, several species within this family are among the most threatened taxa as classified by the International Union for Conservation of Nature (IUCN) Red List of Threatened Species (Van Dijk et al. 2000). Among these, the Crowned River Turtle *Hardella thurjii* (Gray 1831), a large‐sized freshwater species endemic to the Indian subcontinent (Bangladesh, India, and Pakistan) (Ahmed et al. 2021). Initially described as *Emys thurjii*, subsequent taxonomic revisions assigned this species to the monotypic genus *Hardella* Gray, 1870 due to its unique morphological characteristics (Das and Bhupathy 2009). This species is distinguished by its thick, heavy shell, with a weak vertebral keel observed in eastern populations and additional pleural keels in western populations. The species exhibits a dark brown carapace with yellowish bands and a yellow plastron marked by black blotches. The pronounced sexual dimorphism is evident, with females attaining a carapace length of up to 65 cm, nearly three times larger than males (Basu 1998). The distribution of *H. thurjii* spans the northern river systems of the Indian subcontinent, including the Indus, Ganges, and Brahmaputra rivers, with its range extending into Pakistan and Bangladesh (Das and Bhupathy 2009). The species primarily inhabits lentic and slow‐moving water bodies such as ponds and oxbow lakes, with a predominantly herbivorous diet that occasionally includes crustaceans and small fishes (Rashid and Swingland 1997). The reproduction of this species occurs during the dry season, with females laying clutches of 8–19 eggs. Furthermore, the incubation lasts several months, and hatchlings measure 41–46 mm in carapace length (Basu 1998). In addition, the species is threatened by habitat destruction, pollution, and exploitation, and is classified as ‘Endangered’ by the IUCN, underscoring the need for conservation measures, including wetland sanctuaries and captive breeding programs (Das and Bhupathy 2009; Praschag et al. 2007).

Beyond morphological studies, molecular research on *H. thurjii* has focused on partial nuclear and mitochondrial genes to elucidate its phylogenetic relationships within the family Geoemydidae (Honda et al. 2002; Spinks et al. 2004; Le et al. 2007; Praschag et al. 2007; Rohilla and Tiwari 2008; Reid et al. 2011). The recent phylogenomic studies have integrated genetic data from this species to investigate turtle‐archosaur affinities and climate‐driven diversification on continental margins (Fong et al. 2012; Thomson et al. 2021). Additionally, population genetics and forensic studies have utilized DNA sequences of geoemydids, including *H. thurjii* from the Indian subcontinent for species‐level identification (Bhaskar and Mohindra 2018; Kundu, Kumar, Laskar, et al. 2018; Rajpoot et al. 2019; Yadav et al. 2021). Furthermore, the comprehensive mitogenomic data have recently emerged as critical tools for evaluating genetic structures and interspecies variations (Parham et al. 2006; Satoh et al. 2016). Globally, herpetologists have sequenced the complete mitogenomes of several freshwater turtles to refine phylogenetic placement and evolutionary relationships (Zardoya and Meyer 1998; Kumazawa and Nishida 1999; Mindell et al. 1999; Kundu et al. 2019, 2020). However, the complete mitochondrial genome of monotypic *H. thurjii* remains unavailable, limiting insights into its matrilineal relationships within the geoemydids lineage.

In addition to molecular research, habitat dynamics are vital for developing conservation strategies for several threatened vertebrates (Van Teeffelen et al. 2012). Multidisciplinary approaches recommended by the IUCN Species Survival Commission (SSC) Tortoise and Freshwater Turtle Specialist Group (TFTSG) emphasize integrating both ecological and genetic data for achieving precise conservation strategies (McMahon et al. 2011; Kundu et al. 2023; Coelho et al. 2024). However, studies on habitat suitability and the impact of climate change on freshwater turtles, particularly *H. thurjii* , remain limited across its native range. In this regard, species distribution modeling (SDM) has proven valuable for predicting habitat conditions with high precision across spatial and temporal scales (Guisan and Zimmermann 2000; Elith and Leathwick 2009). This approach is also instrumental in uncovering ecological and biogeographical relationships essential for designing and implementing targeted conservation plans (Bellard et al. 2012; Peterson and Soberón 2012; Araújo et al. 2019). Moreover, it will contribute to the formulation and implementation of emergency recovery plans aimed at halting the decline and promoting the restoration of freshwater biodiversity using multidisciplinary approaches (Tickner et al. 2020; Ottoni et al. 2023). Thus, to address these challenges, the present study aims to adopt an integrated approach by (i) generating the complete mitochondrial genome of *H. thurjii* using next‐generation sequencing technologies, (ii) analyzing mitogenomes to reveal genetic structures and variations in comparison with related geoemydid species, (iii) conducting cladistic analyses to determine the matrilineal evolutionary relationships of monotypic *H. thurjii* within major Testudines lineages, and (iv) assessing habitat suitability within its IUCN‐designated range. This research provides a robust framework for understanding the past evolutionary history of *H. thurjii* and its current and future spatial ecological conditions. Such unified efforts offer a comprehensive foundation for addressing the conservation challenges of freshwater turtles both regionally and globally. Moreover, conducting similar studies on other Testudines species would represent a novel and comprehensive approach to bridging existing knowledge gaps and contributing to their global conservation efforts.

## 2 Materials and Methods

### 2.1 Sampling, Study Area, and Occurrence Records

**FIGURE 1:**
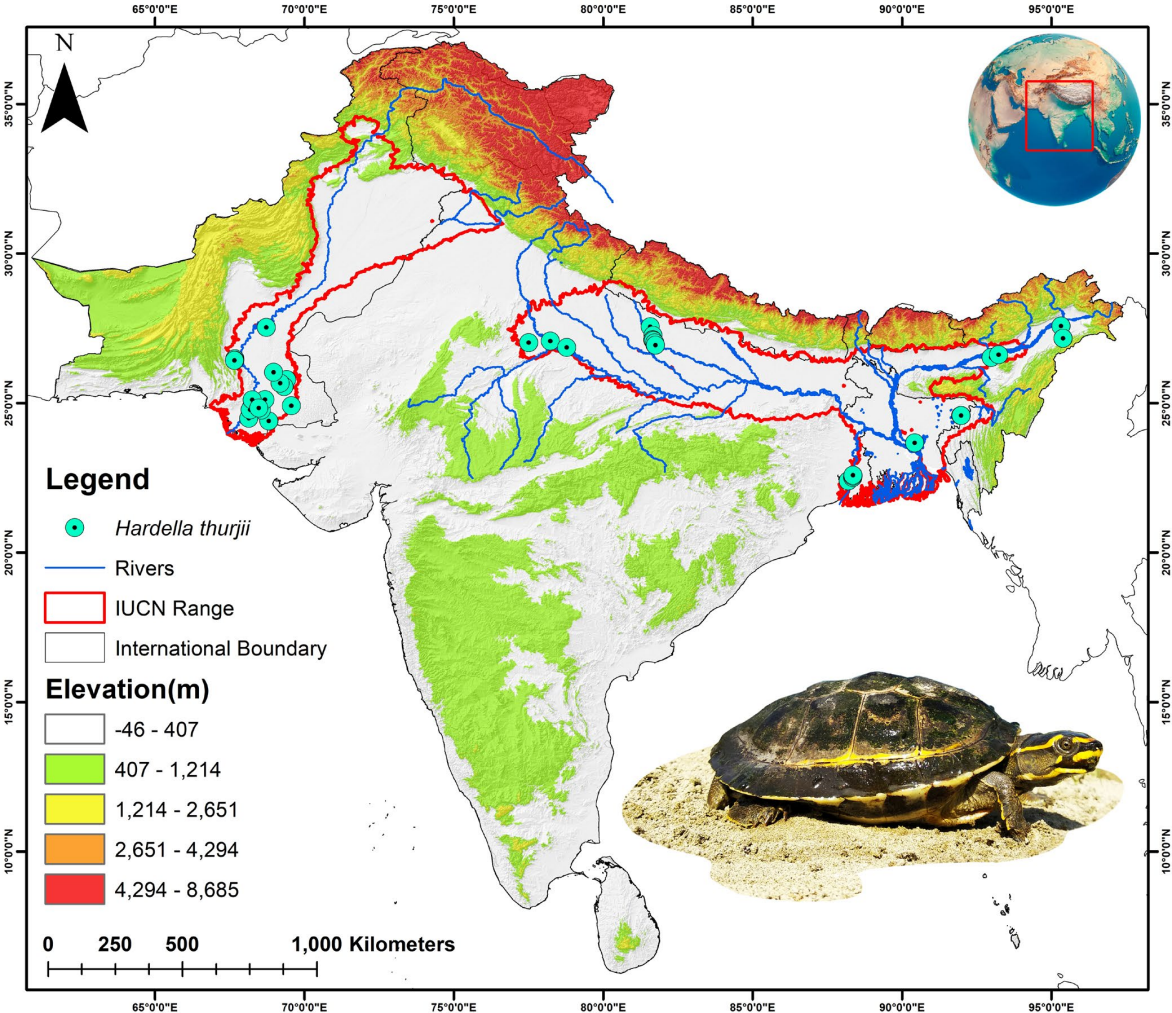
The figure depicts the complete IUCN extant range of the Crowned River Turtle *Hardella thurjii* across the Indian subcontinent, with occurrence points acquired from primary field surveys and secondary sources. The photograph of *H. thurjii* , taken by the fourth author (S.S.).

The specimen of *H. thurjii* was collected from Uttar Pradesh, India, and its identification was confirmed based on key morphological characteristics described in previous literatures (Das 1991; Das and Bhupathy 2009). The blood sample (100 μL) was aseptically collected from the hind limb of the specimen after sedation with 20–30 mg/kg Alfaxalone. Subsequently, the blood sample was preserved in a 1.5 mL EDTA‐containing centrifuge tube and stored at 4°C for further molecular analyses. Furthermore, the entire IUCN‐designated range of *H. thurjii* encompassing the Indus‐Ganges‐Brahmaputra (IGB) River basin was selected as the training area for SDM development (Figure 1) (Ahmed et al. 2021). This training extent is clearly divided into two distinct regions comprising the Eastern (Ganges‐Brahmaputra) and the Western (Indus) ranges. The occurrence points (*n* = 51) were obtained through primary field surveys conducted in Uttar Pradesh by the team of ecologists from the Turtle Survival Alliance Foundation India (TSAFI). Additionally, to achieve a wide array and overall distribution of this species, the study incorporated occurrence points from secondary sources such as GeoCAT (*n* = 48) and available literature (*n* = 35) (Bachman et al. 2011; TTWG Turtle Taxonomy Working Group 2021). However, to ensure an unbiased and robust dataset, specifically, the preserved specimens or captive individuals were deliberately excluded during the aggregation of secondary data. Moreover, the spatial correlations among presence locations were analyzed at a 1 km^2^ resolution using the spatial rarefaction function in SDM Toolbox v2.4 (Brown et al. 2017). This rarefaction scale was selected to align with the raster pixel size and minimize redundancy while reducing the potential for model overfitting, and the final habitat model was run using (*n* = 111) occurrences.

### 2.2 Ethics Statement

No animals were captured from the wild or vouchered during this study. A captive specimen was handled by researchers from TSAFI under the appropriate permission (Principal Chief Conservator of Forests Office, 1451/23–2‐12(G), Lucknow, dated January 5, 2021) granted by the Forest Department, Government of Uttar Pradesh, India. All procedures were conducted in accordance with the ARRIVE 2.0 guidelines for animal research (Percie du Sert et al. 2020; https://arriveguidelines.org).

### 2.3 Mitochondrial DNA Extraction and Next‐Generation Sequencing

The molecular analyses, including mitogenome sequencing, were conducted at Unipath Specialty Laboratory Ltd. (http://www.unipath.in/) in Ahmedabad, India. Mitochondrial DNA was extracted using the Alexgen DNA Kit (Alexius Biosciences, Ahmedabad, Gujarat, India) following the protocol (Ahmed et al. 2021). DNA quantification was performed with a Qubit 4.0 fluorometer to ensure accurate measurements. For sequencing, a paired‐end library was constructed using the QIAseq FX DNA Library Kit (CAT‐180479). DNA fragmentation was achieved with the Covaris M220 Focused Ultrasonicator (Covaris Inc., San Diego, CA, USA), generating smaller fragments for sequencing. These fragments were then subjected to adapter ligation at both ends to enable compatibility with the Illumina sequencing platform. To enhance sequencing efficiency from limited DNA input, high‐fidelity amplification was carried out using the HiFi PCR Master Mix (Takara Bio Inc., Kusatsu, Shiga, Japan). The quality and integrity of the prepared libraries were assessed using the TapeStation 4150 (Agilent Technologies, Santa Clara, CA, USA) with High Sensitivity D1000 ScreenTape, following the manufacturer's guidelines. Based on TapeStation results, DNA concentration and fragment size distribution were confirmed. Finally, the sequencing libraries were processed on the Illumina NovaSeq 6000 platform (Illumina, San Diego, CA, USA) for cluster generation and high‐throughput sequencing.

### 2.4 Mitogenome Assembly, Annotation, and Submission

The high‐quality paired‐end reads (~25 million) were assembled and annotated using Geneious Prime v2023.0.1 (Kearse et al. 2012). Gene boundaries and strand orientations were verified through the MITOS Galaxy web server (http://mitos.bioinf.uni‐leipzig.de) and MitoAnnotator (http://mitofish.aori.u‐tokyo.ac.jp/annotation/input/) (Iwasaki et al. 2013; Bernt et al. 2013). To ensure the accuracy of protein‐coding genes (PCGs), their amino acid sequences were validated against the vertebrate mitochondrial genetic code using the ORF Finder tool (https://www.ncbi.nlm.nih.gov/orffinder/). The initiation and termination codons were identified by referencing mitochondrial genomes within the subfamily Batagurinae ( *Batagur kachuga* (Gray, 1831): MZ562559, *Geoclemys hamiltonii* (Gray, 1831): OP344485, *Pangshura sylhetensis* Jerdon, 1870: MK580979). The newly assembled mitogenome (Accession No. PP336441) was submitted to GenBank using the Sequin submission tool, accompanied by a gene feature file specifying precise boundaries and strand orientations.

### 2.5 Mitogenome Characterization and Evaluation of Control Region

The circular representation of the *H. thurjii* mitogenome was generated using the MitoAnnotator web server, with intergenic spacers and overlapping regions manually annotated. The sizes and nucleotide compositions of PCGs, ribosomal RNA (rRNA) genes, and transfer RNA (tRNA) genes were analyzed using MEGA 11 (Tamura et al. 2021). To assess nucleotide composition bias, the base composition skew was calculated using the formulas: AT‐skew = (A − T)/(A + T) and GC‐skew = (G − C)/(G + C), following the methodology (Perna and Kocher 1995). The control region (CR) was examined to identify structural domains based on previous studies, with comparative analyses conducted across nine other species within the subfamily Batagurinae. However, *Batagur dhongoka* (Gray, 1832) (Accession number MZ242096) was excluded due to the absence of the non‐coding AT‐rich region (Kundu et al. 2023). Furthermore, tandem repeats within the CR were identified using the Tandem Repeats Finder tool (https://tandem.bu.edu/trf/trf.html) (Benson 1999).

### 2.6 Dataset Construction and Phylogenetic Analyses

To explore the evolutionary relationships among geoemydid turtles, the complete mitogenomes of 47 species were retrieved from GenBank (Table S1). Additionally, to provide a broader cladistic context within the suborder Cryptodira, nine representative mitogenomes were randomly selected from different turtle families, including Trionychidae, Carettochelyidae, Kinosternidae, Chelydridae, Dermochelyidae, Cheloniidae, Testudinidae, Platysternidae, and Emydidae. Further, the mitogenomes of three families within the suborder Pleurodira (Podocnemididae, Pelomedusidae, and Chelidae) were incorporated as an outgroup. A concatenated dataset comprising 13 PCG was generated using iTaxoTools 0.1, and the optimal nucleotide substitution model (GTR + G + I) was identified through PartitionFinder 2, accessed via the CIPRES Science Gateway v3.3 (Miller et al. 2015; Lanfear et al. 2017; Vences et al. 2021). The phylogenetic analyses were conducted using both Bayesian Inference (BI) and Maximum‐Likelihood (ML) methods to reconstruct evolutionary relationships within Testudines. For the Bayesian inference, MrBayes 3.1.2 (Ronquist and Huelsenbeck 2003) was employed with the following parameters: nst = 6, one cold chain, and three heated Metropolis‐coupled Markov chain Monte Carlo (MCMC) chains. The analysis was executed for 10,000,000 generations, with trees sampled every 100 generations, and the first 25% of samples discarded as burn‐in. The ML topology was inferred using the PhyML 3.0 web server with 1000 bootstrap replicates, following standard procedures (Guindon et al. 2010). Both Bayesian and ML‐generated phylogenies were visualized and refined using the Interactive Tree of Life (iTOL) v4 web server (https://itol.embl.de/login.cgi) (Letunic and Bork 2021) to enhance interpretability.

### 2.7 Selection of Model Covariates

To model the habitat suitability of *H. thurjii* , bioclimatic, anthropogenic, habitat, and topographic variables were selected based on established methodologies (Peterson and Soberón 2012). The 19 standard bioclimatic variables commonly utilized in SDM were obtained from the WorldClim database (https://www.worldclim.org/) (Fick and Hijmans 2017). Additionally, considering the aquatic nature of this turtle species, the habitat variable of Euclidean distance to water bodies was incorporated, derived from the global Land Use Land Cover (LULC) data provided by the ESRI Sentinel−2 10‐Meter Land Use/Land Cover dataset available on the Living Atlas platform (https://livingatlas.arcgis.com/landcover/) (Karra et al. 2021). This dataset was processed into a continuous raster format using the Euclidean distance function in ArcGIS 10.6 to assess habitat proximity (Abedin et al. 2025). Furthermore, the topographic variable elevation was extracted using 90‐m Shuttle Radar Topography Mission (SRTM) data (http://srtm.csi.cgiar.org/srtmdata/). The Global Human Footprint Dataset was utilized as an anthropogenic predictor to evaluate the Human Influence Index (HII) and understand the extent of human impact on the target species (Wildlife Conservation Society 2005). Moreover, all spatial variables were standardized to a resolution of 30 arcseconds (~1 km^2^) using the Spatial Analyst extension in ArcGIS 10.6. To ensure robustness in the analysis, spatial multicollinearity testing was performed using the SAHM (Software for Assisted Habitat Modeling) package in VisTrails software (Morisette et al. 2013). Variables with a Pearson correlation coefficient (r) exceeding 0.8 were excluded from further analysis to reduce redundancy (Warren et al. 2010) (Figure S1).

Furthermore, to assess the potential impacts of climate change, the study evaluated future scenarios under two Shared Socioeconomic Pathways (SSPs): SSP245 and SSP585, for the periods 2041–2060 and 2061–2080. Climate projections were sourced from the HadGEM3‐GC31 LL model, part of the Coupled Model Intercomparison Project Phase 6 (CMIP6) (Li et al. 2023; Gautam and Shany 2024). Consequently, the nonclimatic variables were kept constant in future climate analyses to isolate the impacts of climatic changes on the species' distribution, restricting projections to ecologically relevant areas for *H. thurjii* (Allen et al. 2024).

### 2.8 Model Configuration and Evaluation

The habitat modeling in this study employed an ensemble approach that integrated multiple algorithms to construct a comprehensive model that represents a wide array of information and robustness for the target species. Thus, by combining the distinct strengths of each algorithm, this method effectively captures diverse factors influencing species distribution, enhancing prediction accuracy and reliability (Hao et al. 2020). The five selected algorithms—Boosted Regression Tree (BRT), Multivariate Adaptive Regression Splines (MARS), Generalized Linear Model (GLM), Maximum Entropy (MaxEnt), and Random Forest (RF)—were chosen for their ability to account for varied species‐environment interactions (Guisan et al. 2007; Elith and Leathwick 2009; Miller 2010). These models were implemented using the SAHM package in VisTrails software; the models produced probability maps ranging from ‘0’ (least suitable) to ‘1’ (most suitable), with binary maps generated using the minimum training presence threshold (Talbert and Talbert 2012; Morisette et al. 2013). Additionally, an ensemble count map was created to assess agreement across models, with each pixel indicating the degree of model agreement. The model performance was evaluated using the Area Under the Curve (AUC) metric, with a threshold of 0.75 set for validation (Lavazza et al. 2023). Moreover, to ensure robustness and assessment of the models, a few performance metrics such as AUC, True Skill Statistic (TSS), Cohen's Kappa, Proportion Correctly Classified (PCC), specificity, and sensitivity were calculated across training and cross‐validation datasets (*n* = 10), confirming the reliability of the final model for predicting species distribution (Cohen 1968; Allouche et al. 2006; Phillips and Elith 2010; Jiménez‐Valverde et al. 2013).

### 2.9 Evaluation of Habitat Quality and Shape Geometry

The qualitative and geometric characteristics of suitable habitat patches for *H. thurjii* within the eastern and western ranges were evaluated under current and projected future climatic scenarios to enable a comparative analysis. For this purpose, class‐level metrics were assessed using FRAGSTATS software version 4.2.1 (McGarigal and Marks 1995), a widely utilized tool in landscape ecology and environmental management. This software provides an extensive suite of metrics to analyze spatial patterns, offering valuable insights into the structure and composition of landscapes (Hesselbarth et al. 2019). The key metrics used in this study included the number of patches (NP), largest patch index (LPI), patch density (PD), total edge (TE), aggregate index (AI), and landscape shape index (LSI). While NP, PD, TE, and LPI provided detailed insights into patch size, edge characteristics, and density, the LSI metric assessed shape complexity, and the AI quantified patch aggregation, reflecting their spatial proximity and clustering within the landscape.

## 3 Results

### 3.1 Mitogenomic Structure and Variations

**FIGURE 2:**
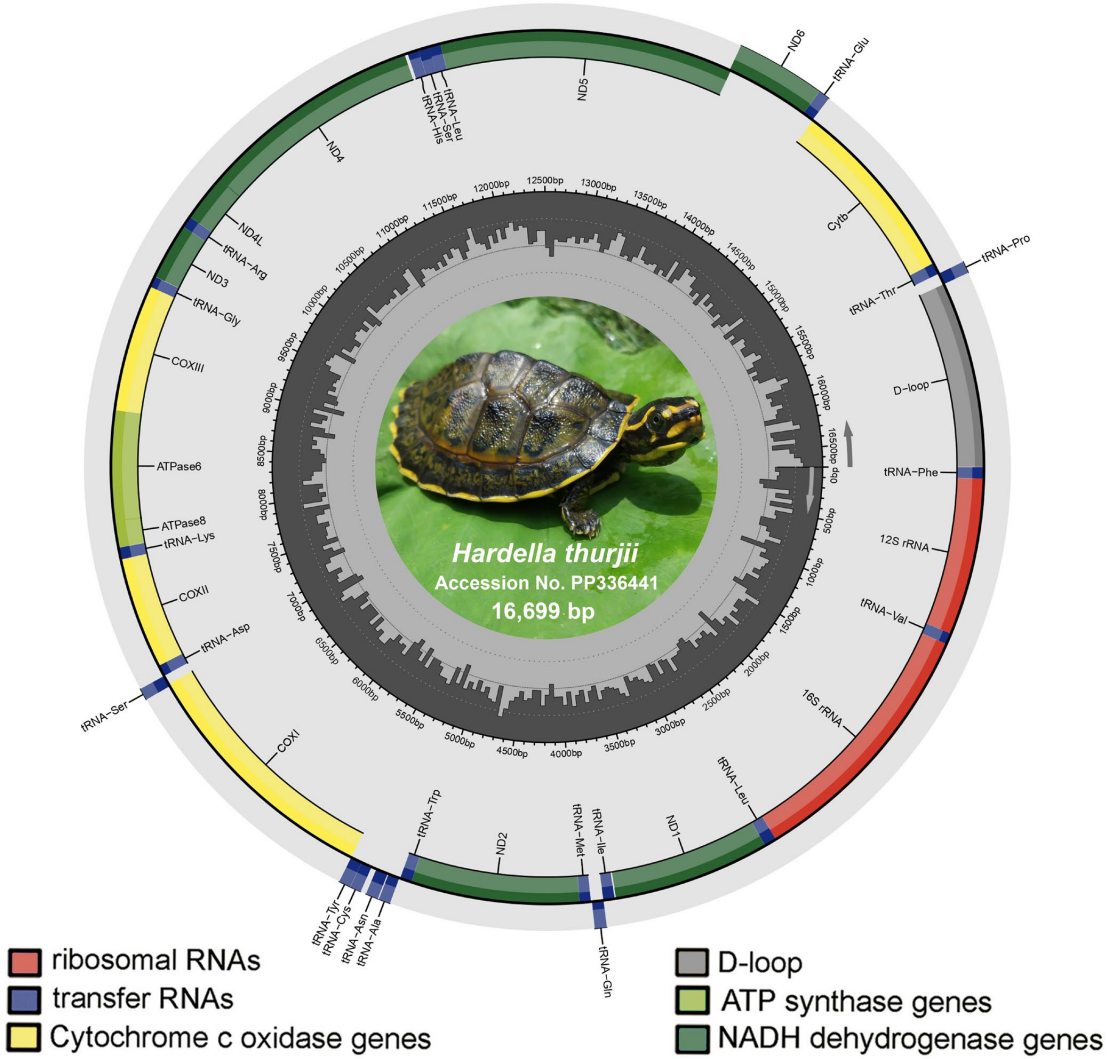
The circular complete mitogenome of *H. thurjii* was visualized using the MitoAnnotator online web server. Genes encoded on the heavy and light strands are displayed inside and outside the circular gene map, respectively. Different gene groups, including PCGs, rRNA, tRNA, and the non‐coding control region, are represented by distinct colors, indicating their strand encoding and boundaries. A photograph of a representative species was taken by the second author (A.S.).

This present research characterizes the mitogenome of *H. thurjii* , to elucidate its genetic structure and variations. The mitogenome (16,699 bp) was sequenced and submitted in GenBank under accession number PP336441. The circular mitogenome comprises 13 PCGs, 22 tRNAs, two rRNAs, and a major non‐coding AT‐rich CR. Among these, nine genes (including *nad6* and eight tRNAs) are located on the light strand, while the other 28 genes are positioned on the heavy strand (Figure 2; Figure S2; Table 1; Table S1). Within the lineage of the subfamily Batagurinae, the mitogenomic lengths are lowest (16,397 bp) in *Batagur borneoensis* (Schlegel & Müller 1845) and highest (17,588 bp) in *Orlitia borneensis* Gray, 1873. The nucleotide composition of the *H. thurjii* mitogenome was A + T biased (59.04%), and the AT skew and GC skew were 0.141 and −0.350 respectively. The total length of PCGs was 11,382 bp (68.15%); rRNAs were 2551 bp (15.27%); tRNAs were 1565 bp (9.37%); and CR was 1149 bp (6.87%) in *H. thurjii* mitogenome. A total of 12 overlapping regions (total length of 47 bp) were found in *H. thurjii* mitogenome, with the longest region (13 bp) between Cytochrome c oxidase subunit I (*cox1*) and tRNA‐Serine (*trnS2*). Following this, the next longest overlap region (10 bp) was found between ATP synthase membrane subunit 8 (*atp8*) and ATP synthase membrane subunit 6 (*atp6*) genes. Further, a total of 11 intergenic spacer regions (total length of 99 bp) were identified in *H. thurjii* , with the longest region (31 bp) between tRNA‐Asparagine (*trnN*) and tRNA‐Cysteine (*trnC*). Following this, the next longest intergenic spacer region (19 bp) was found between tRNA‐Valine (*trnV*) and large ribosomal RNA (*rrnL*) as well as between NADH dehydrogenase subunit 4 (*nad4*) and tRNA‐Histidine (*trnH*) genes. The majority of the PCGs in *H. thurjii* begin with the ATG initiation codon, except for the *cox1* gene, which utilizes GTG. The distribution of initiation codons among PCGs is largely consistent with other species within the Batagurinae subfamily. Regarding termination codons, five PCGs end with the TAA stop codon, whereas others terminate with alternative codons (AGG, TAG, and AGA) or incomplete stop codons (T– and TA‐). Both ribosomal RNA genes (*rrnS* and *rrnL*) are located on the heavy strand, similar to other Testudines species. Among the 22 transfer RNA (tRNA) genes, 14 are positioned on the heavy strand, while the remaining eight are located on the light strand, each with distinct anticodons.

**TABLE 1:** List of annotated mitochondrial genes of *H. thurjii* .

Gene	Direction	Start	Stop	Size	Anti‐codon	Start codon	Stop codon	Intergenic nucleotides
*trnF*	+	1	70	70	GAA	—	—	−1
*rrnS*	+	70	1039	970	—			−1
*trnV*	+	1039	1109	71	TAC	—	—	19
*rrnL*	+	1129	2709	1581	—			0
*trnL2*	+	2710	2786	77	TAA	—	—	0
*nad1*	+	2787	3755	969	—	ATG	TAA	7
*trnI*	+	3763	3833	71	GAT	—	—	−2
*trnQ*	−	3903	3832	70	TTG	—	—	5
*trnM*	+	3909	3978	70	CAT	—	—	0
*nad2*	+	3979	5017	1039	—	ATG	T‐—	−1
*trnW*	+	5017	5089	73	TCA	—	—	5
*trnA*	−	5164	5095	68	TGC	—	—	5
*trnN*	−	5244	5170	73	GTT	—	—	31
*trnC*	−	5342	5276	65	GCA	—	—	−1
*trnY*	−	5413	5342	70	GTA	—	—	1
*cox1*	+	5415	6965	1551	—	GTG	AGG	−13
*trnS2*	−	7024	6953	70	TGA	—	—	−1
*trnD*	+	7024	7094	71	GTC	—	—	0
*cox2*	+	7095	7781	687	—	ATG	TAA	0
*trnK*	+	7782	7855	74	TTT	—	—	1
*atp8*	+	7857	8024	168	—	ATG	TAA	−10
*atp6*	+	8015	8697	683	—	ATG	TA—	0
*cox3*	+	8698	9481	784	—	ATG	T‐—	−1
*trnG*	+	9481	9549	69	TCC	—	—	1
*nad3*	+	9551	9899	349	—	ATG	T‐—	0
*trnR*	+	9900	9970	71	TCG	—	—	0
*nad4L*	+	9971	10,267	297	—	ATG	TAA	−7
*nad4*	+	10,261	11,637	1377	—	ATG	TAA	19
*trnH*	+	11,657	11,725	69	GTG	—	—	0
*trnS1*	+	11,726	11,792	67	GCT	—	—	−1
*trnL1*	+	11,792	11,864	73	TAG	—	—	0
*nad5*	+	11,865	13,676	1812	—	ATG	TAG	−8
*nad6*	−	14,190	13,669	522	—	ATG	AGA	0
*trnE*	−	14,258	14,191	66	TTC	—	—	5
*cytb*	+	14,264	15,407	1144	—	ATG	T‐—	0
*trnT*	+	15,408	15,479	72	TGT	—	—	0
*trnP*	−	15,550	15,480	69	TGG	—	—	0
*CR*		15,551	16,699	1149	—	—	—	—

### 3.2 Characteristics of Non‐Coding Control Region

**FIGURE 3:**
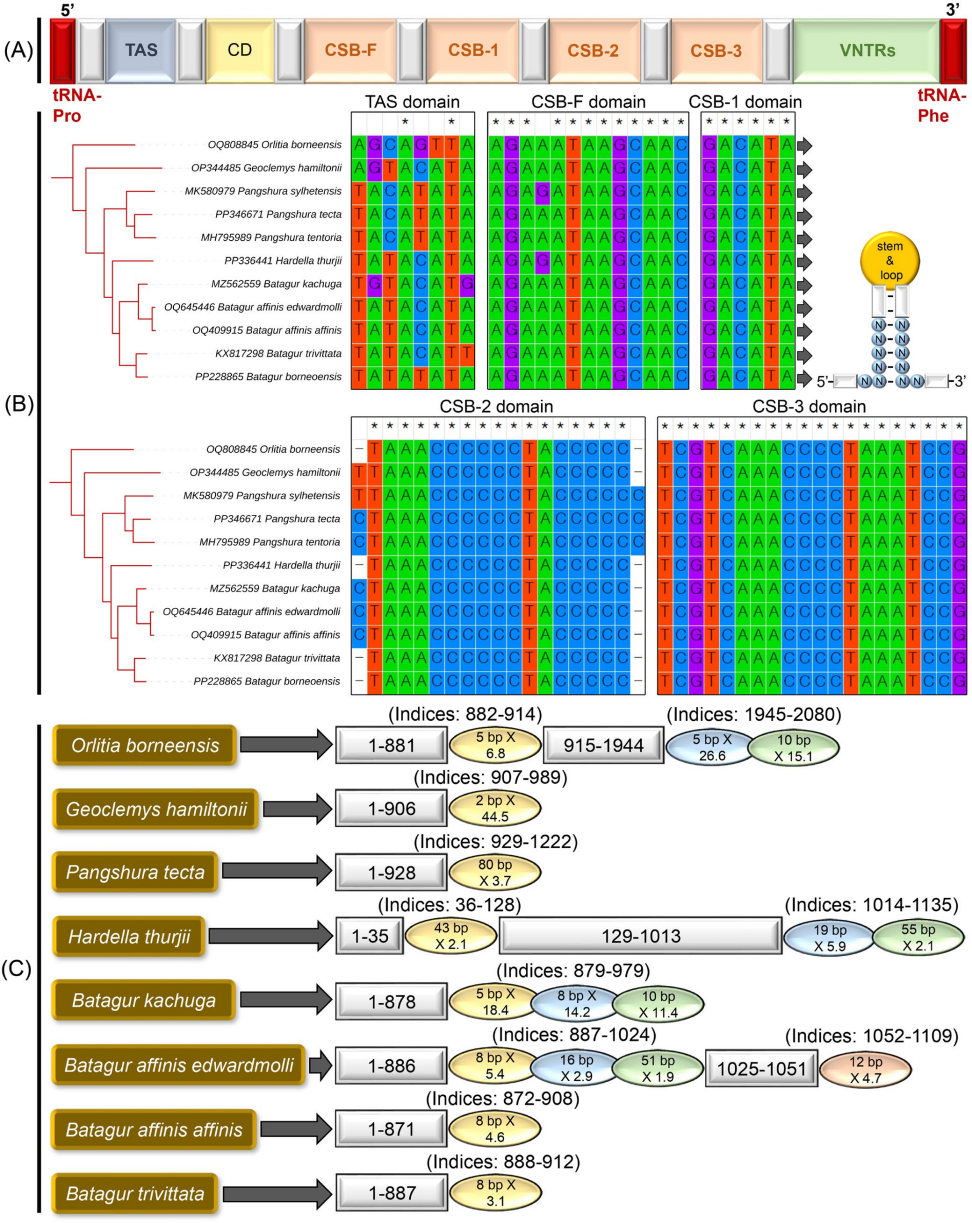
(A) Generalized linear representation of the control region (CR) of the Testudines mitogenome, (B) Structural organization of the conserved domain across Batagurinae taxa, and (C) Location and copy number of tandem repeats are illustrated using colored oval shapes, as predicted by the Tandem Repeats Finder online tool (https://tandem.bu.edu/trf/trf.html).

The CR of *H. thurjii* is structurally composed of three functional domains: the termination‐associated sequence (TAS), the central conserved domain (CD), and the conserved sequence block (CSB) (Figure 3). High nucleotide conservation was observed in both the TAS and four distinct CSB domains (CSB‐F, CSB‐1, CSB‐2, and CSB‐3). Among these, CSB‐3 represents the longest conserved region (20 bp), followed by CSB‐2 (17 bp), CSB‐F (13 bp), and CSB‐1 (6 bp). A unique structural feature was identified in *H. thurjii* and other Batagurinae species, attributed to the presence of the conserved motif (GACATA) within the CSB‐1 domain. These stem‐loop structures play a crucial role in regulating mitochondrial transcription and replication. Additionally, a two‐base pair nucleotide deletion was detected within the CSB‐2 domain of *H. thurjii* and other Batagurinae species, including *O. borneensis* , *Batagur trivittata* (Duméril & Bibron, 1835), and *B. borneoensis* (Table S1). Remarkably, Batagurinae species exhibit a distinct pattern of variable number tandem repeats (VNTRs) in their control regions. The monotypic *H. thurjii* contains three different consensus tandem repeats (43 bp > 2 times, 19 bp > 5 times, and 55 bp > 2 times) in its CR. Similarly, *O. borneensis* and *B. kachuga* also exhibit three types of consensus tandem repeats, whereas the remaining four Batagurinae species display only a single consensus tandem repeat.

### 3.3 Phylogenetic Placement and Major Evolutionary Relationship

**FIGURE 4:**
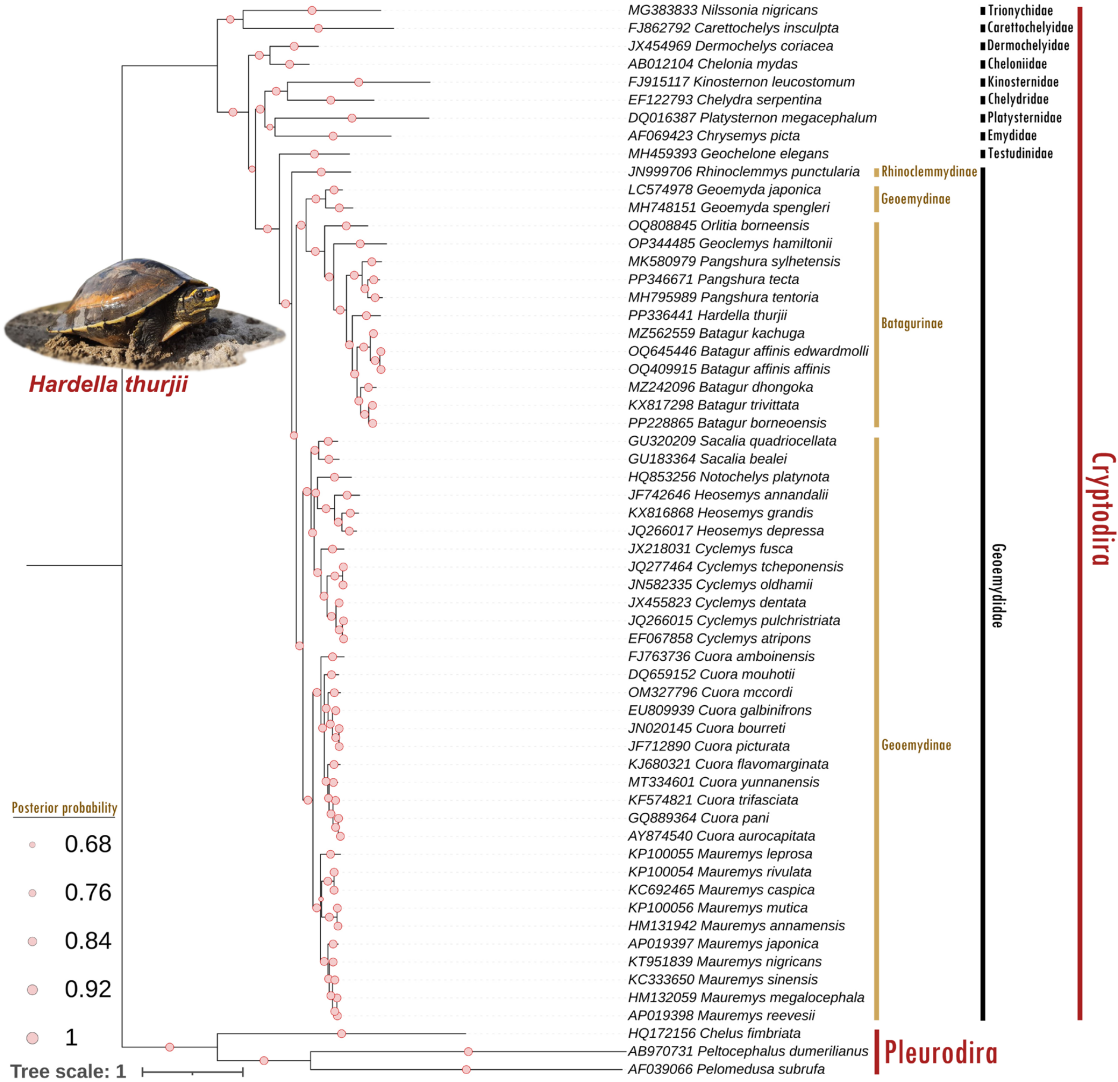
The unified Bayesian inference (BI) phylogenetic tree, constructed using the concatenated nucleotide sequences of 13 PCGs illustrates the matrilineal evolutionary relationships of Testudines. The analysis supports a sister relationship between *H. thurjii* and other *Batagur* species. BA posterior probability values are represented by light blue circular dots of varying sizes superimposed on each node. Photograph of representative species was taken by Sreeparna Dutta, TSAFI.

The BA phylogeny effectively delineated all Testudines species into their respective family and subfamily groups with strong posterior probability support, utilizing 13 concatenated protein‐coding genes (PCGs) (Figure 4). Within Cryptodira, all turtles exhibited monophyletic clustering, underscoring the close evolutionary relationship between geoemydids and land tortoises (family Testudinidae) rather than other freshwater and marine lineages. The present mitogenomic phylogeny clearly distinguished *H. thurjii* from other Batagurinae species while confirming its sister relationship with the *Batagur* congeners. Overall, the Batagurinae members including *Batagur* Gray, 1856, *Geoclemys* Gray, 1856, *Hardella*, *Pangshura* Gray, 1856, and *Orlitia* Gray, 1873 formed a well‐supported monophyletic group in the mitogenomic phylogeny. Conversely, within the Geoemydinae subfamily, *Geoemyda japonica* Fan, 1931 and *Geoemyda spengleri* (Gmelin, 1789) clustered separately from other extant taxa, forming a paraphyletic group. The ML topology mirrored this clade arrangement, consistently supporting the phylogenetic structure of geoemydid turtles with high bootstrap values at each node (Figure S3).

### 3.4 Model Performance and Identification of Habitat Suitability

**FIGURE 5:**
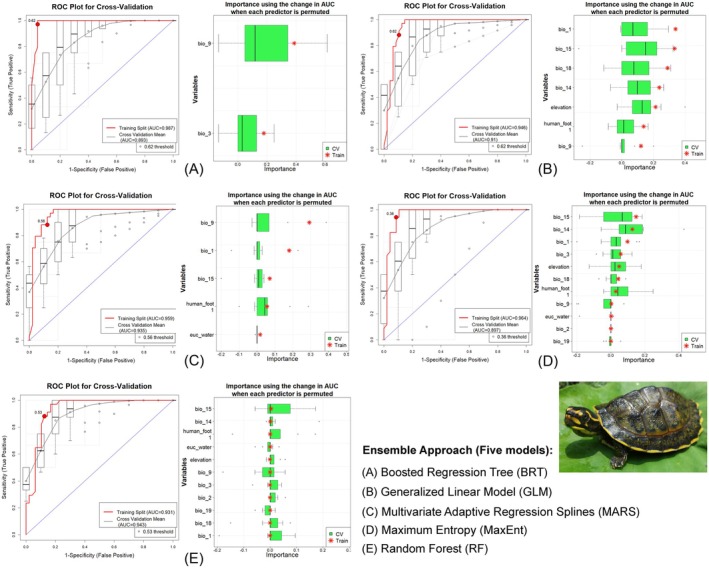
Model evaluation plots showing the average training ROC of both training and cross‐validation (CV) and the predictors chosen by the model for the replicate runs under five models.

The ensemble model developed for the target species yielded good performance, with all models exceeding the AUC threshold of 0.75 in both training and cross‐validation datasets (Figure 5 and Table 2). Specifically, the training AUC ranged from 0.931 to 0.987, while cross‐validation AUC values varied between 0.893 and 0.943. The highest ΔAUC of 0.094 was observed in the BRT model, while the lowest ΔAUC (0.012) occurred in the RF model. Additionally, other evaluation metrics such as PCC, TSS, Cohen's Kappa, specificity, and sensitivity yielded favorable outcomes in both training and cross‐validation runs, thus further confirming the robustness of the model predictions. The ensemble modeling approach revealed that the Mean Temperature of the Driest Quarter (bio_9) emerged as the most significant predictor, contributing 19.51% to the distribution model for the target species (Figure 5, Table 3). In addition to this, the annual mean temperature (bio_1) was identified as one of the key contributing variables, accounting for 16.64% of the total contribution to the model. Furthermore, the Euclidean distance to water (euc_water) was also found to be a relevant factor with a contribution of 5.10% to the prediction of the model. Moreover, the anthropogenic variable Human Influence Index (Human_foot1) was observed to contribute 9.75% to the distribution, thus underscoring its importance in the habitat suitability analysis.

**TABLE 2:** Model fit metrics for each of the participating modeling methods and for the final ensemble model for estimation of habitat suitability of *H. thurjii* .

Model	Dataset	AUC	ΔAUC	PCC	TSS	Kappa	Specificity	Sensitivity
BRT	Train	0.987	0.094	96.3	0.929	0.925	0.958	0.971
	CV	0.893		84.2	0.692	0.681	0.85	0.842
GLM	Train	0.946	0.036	89	0.778	0.775	0.896	0.882
	CV	0.91		85.3	0.7	0.699	0.85	0.85
MARS	Train	0.959	0.024	87.8	0.757	0.751	0.875	0.882
	CV	0.935		84.4	0.678	0.672	0.87	0.808
MaxEnt	Train	0.964	0.067	92.6	0.856	0.849	0.915	0.941
	CV	0.897		85.5	0.702	0.7	0.885	0.817
RF	Train	0.931	0.012	87.8	0.757	0.751	0.875	0.882
	CV	0.943		83	0.655	0.653	0.83	0.825

*Note:* A total of five model algorithms were used with the threshold of < 0.75 AUC score. The models were Maximum Entropy (MaxEnt), Random Forest (RF), Boosted Regression Tree (BRT), Generalized Linear Model (GLM), and Multivariate Adaptive Regression Splines (MARS).

Abbreviations: ΔAUC, Change in Area under curve (Training—Cross Validation; AUC, Area under Curve; PCC, Proportion Correctly Classified; TSS, True Skill Statistic).

**TABLE 3:** The mean percentage contribution of the covariates generated from the final model for *H. thurjii* .

Variable	Abbreviation	BRT	GLM	MARS	MAXENT	RF	*μ* (mean)	*μ* (mean) %
Annual mean temperature	bio_1	0.000	0.340	0.179	0.097	0.056	0.135	16.64
Precipitation of driest month	bio_14	0.000	0.237	0.000	0.126	0.019	0.076	9.44
Precipitation seasonality	bio_15	0.000	0.334	0.070	0.148	0.095	0.130	16.02
Precipitation of warmest quarter	bio_18	0.000	0.289	0.000	0.042	0.056	0.078	9.59
Precipitation of coldest quarter	bio_19	0.000	0.000	0.000	0.000	0.019	0.004	0.48
Mean diurnal range	bio_2	0.000	0.000	0.000	0.000	0.029	0.006	0.71
Isothermality	bio_3	0.178	0.000	0.000	0.037	0.010	0.045	5.56
Mean temperature of driest quarter	bio_9	0.387	0.121	0.222	0.001	0.057	0.158	19.51
Elevation	elevation	0.000	0.214	0.000	0.040	0.037	0.058	7.20
Euclidean distance to water	euc_water	0.000	0.000	0.047	0.059	0.100	0.041	5.10
Human influence index	human_foot1	0.000	0.138	0.056	0.029	0.171	0.079	9.75

**FIGURE 6:**
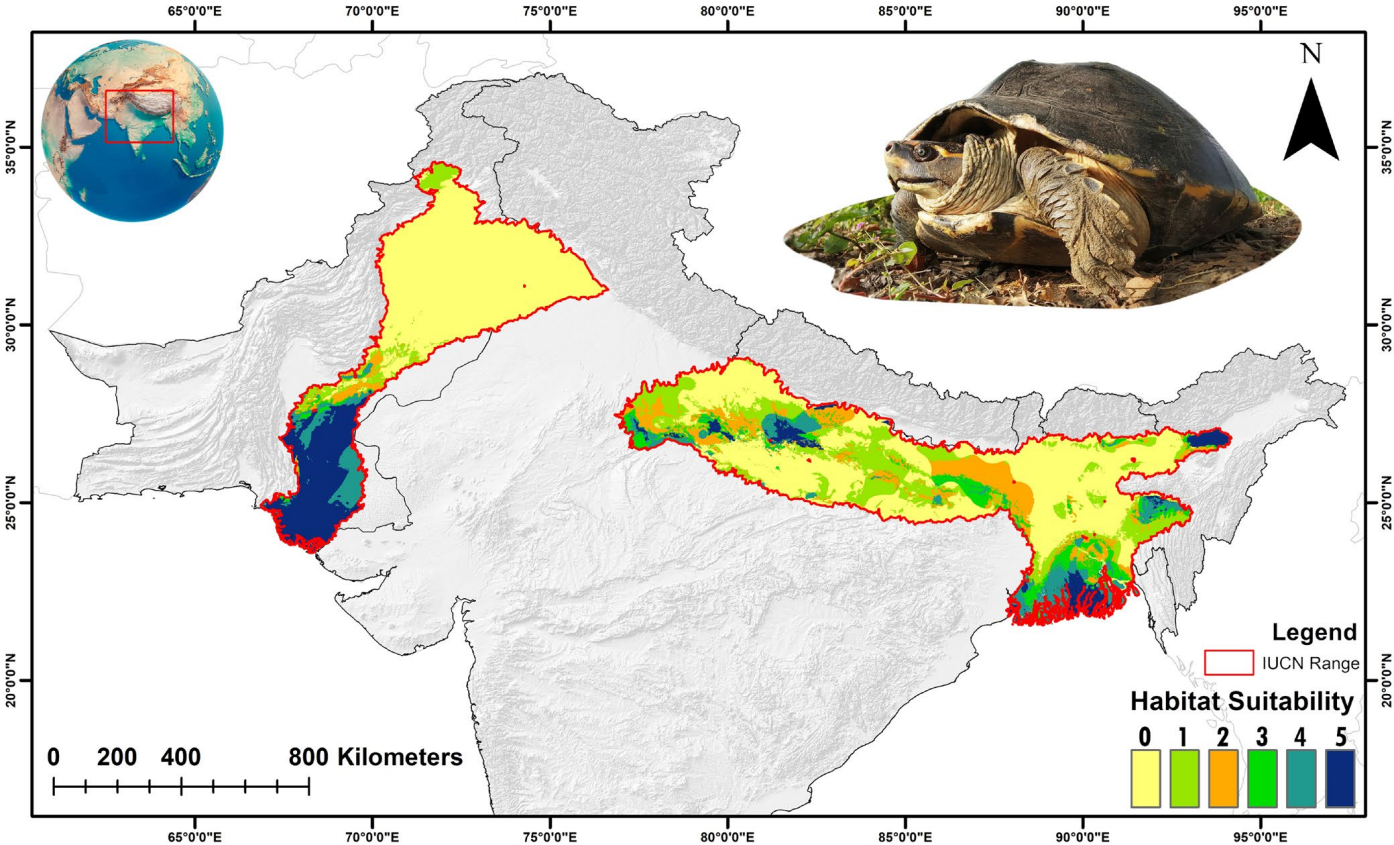
Present suitable habitats for *H. thurjii* in the study area, with habitat suitability ranging from low to high suitability. The photograph of representative species was taken by the fourth author (S.S.).

**FIGURE 7:**
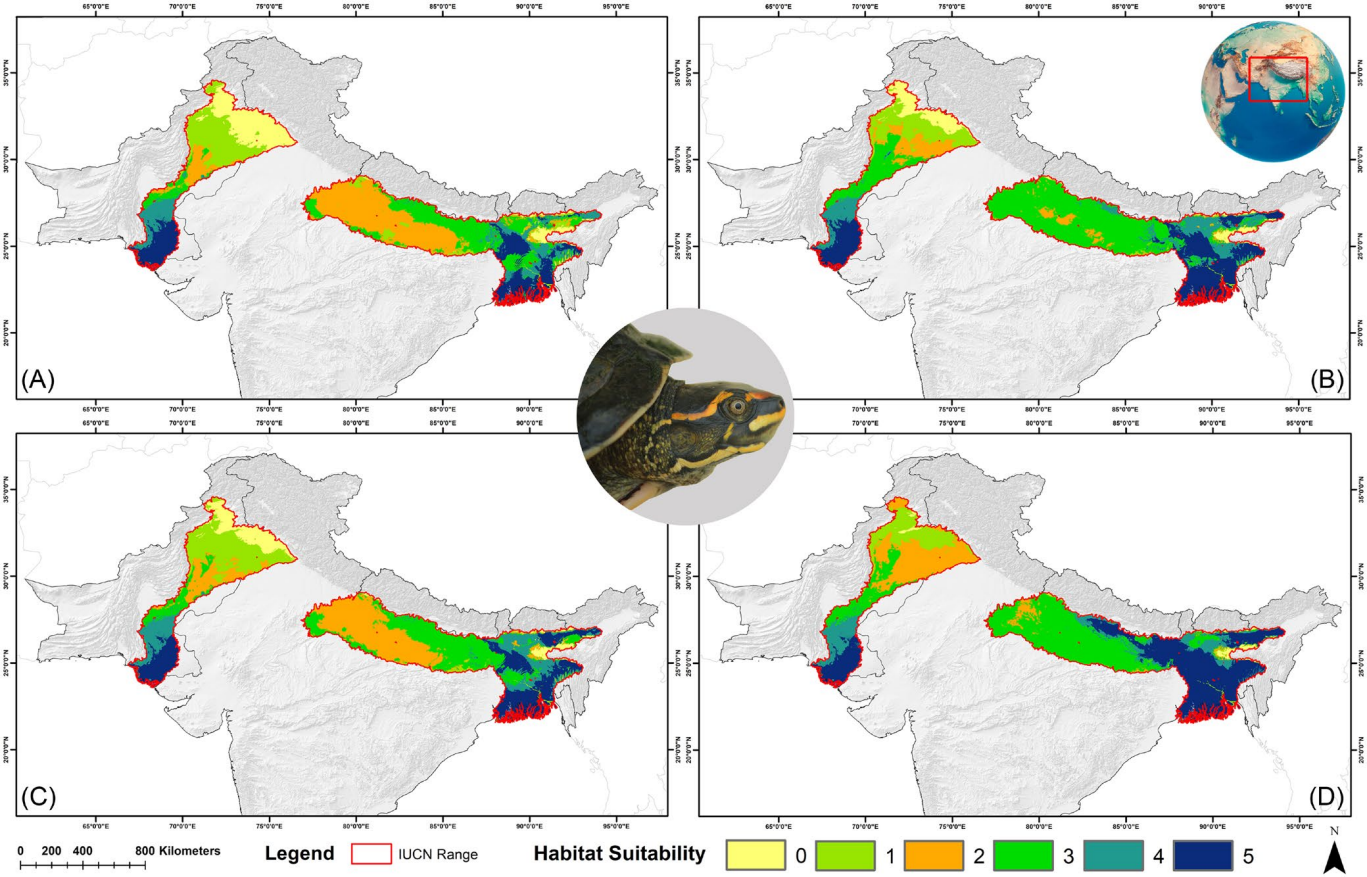
Map showing habitat suitability for *H. thurjii* across its entire IUCN range under future climate change scenarios. The subfigures represent different Shared Socioeconomic Pathways (SSPs) and timeframes: (A) SSP245 (2041–2060), (B) SSP245 (2061–2080), (C) SSP585 (2041–2060), and (D) SSP585 (2061–2080).

The model identified a total of 110,490 km^2^. as suitable habitat for *H. thurjii* across both ranges in the present scenario (Figure 6, Table 4). This suitable area represents a mere 10.32% of the vast IUCN‐designated range of 1,070,448 km^2^. within the IGB River Basin. Furthermore, of the delineated suitable area, the eastern range encompassed 35,757 km^2^., while the western range accounted for 83,723 km^2^. Moreover, the projections under future climatic scenarios revealed intriguing trends, with the overall habitat suitability for *H. thurjii* across its IUCN‐designated range increasing by over 32.38% compared to the present scenario (Figure 7, Table 4). The most significant increases in suitability were observed in the 2061–2080 timeframe under both the SSP245 and SSP585 scenarios, with increases of 87.75% and 118.32%, respectively. However, the eastern and western ranges demonstrated contrasting responses to the future climate scenarios within their respective areas. Notably, the eastern range saw a substantial increase of 183.64% in habitat suitability across all future climatic scenarios compared to the current situation (Figure 7, Table 4). On the contrary, the western range experienced a decline in habitat suitability, with reductions ranging from 27.27% to 38.92% under the future climatic scenarios from the present.

**TABLE 4:** The suitable areas (in km^2^) for *H. thurjii* in Eastern and Western range in present and future climatic scenarios.

Scenario	Eastern	Western	Total area (in km^2^)
Present	35,767	83,723	119,490
SSP245 (2041–2060)	101,453	56,723	158,176
SSP245 (2061–2080)	163,450	60,889	224,339
SSP585 (2041–2060)	131,300	53,235	184,535
SSP585 (2061–2080)	209,737	51,134	260,871

### 3.5 Evaluation of Habitat Fragmentation

**FIGURE 8:**
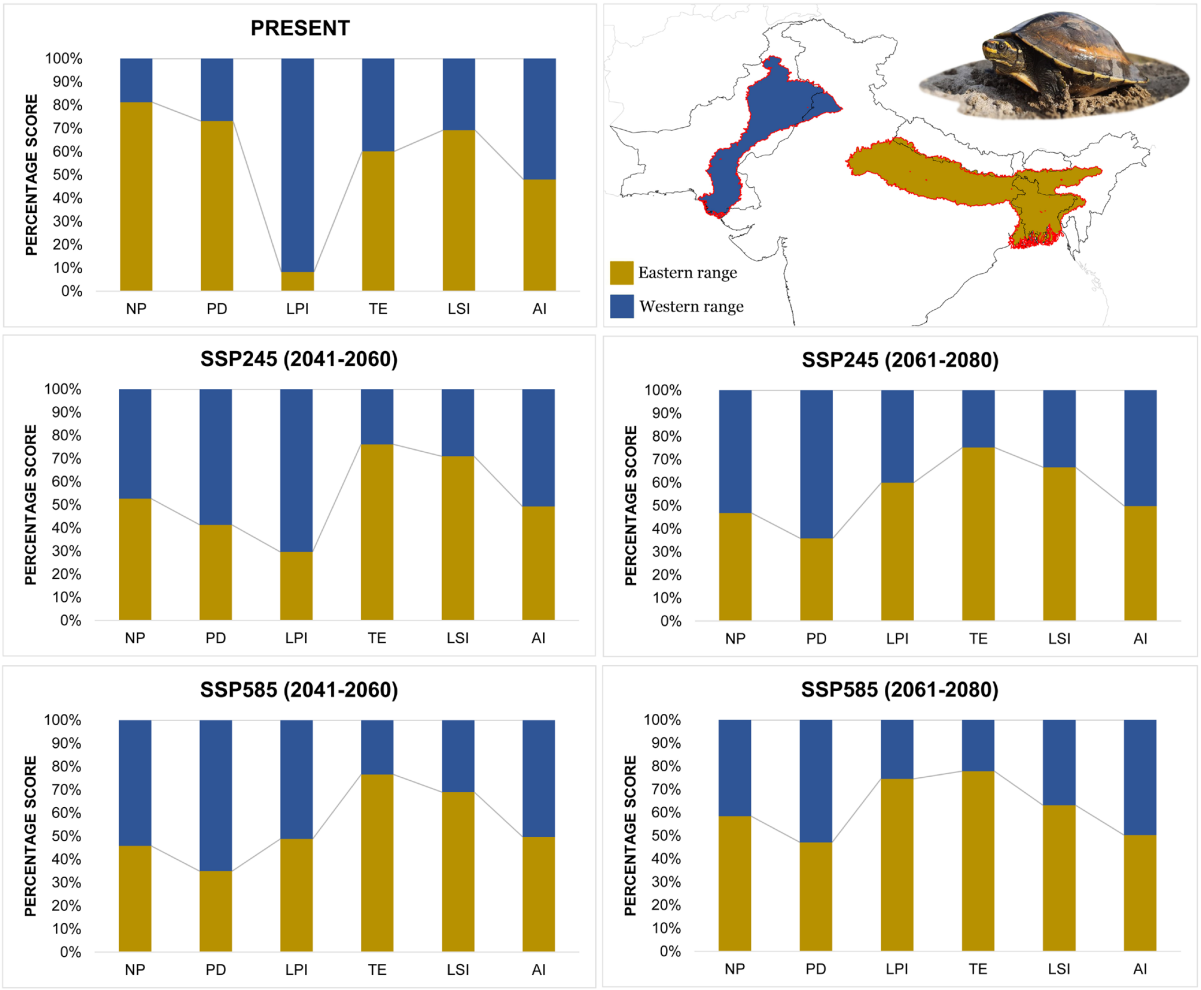
Percentage stack of class‐level metrics used for habitat quality assessment of *H. thurjii* in the Indus River basin and Ganges‐Brahmaputra River Basin. The values indicate scores for various indices: AI, aggregation index; LPI, largest patch index; LSI, landscape shape index; NP, number of patches; PD, patch density; TE, total edge.

The assessment of habitat quality and shape geometry yielded intriguing results for both ranges of the studied species. Specifically, in the eastern range, the number of suitable patches increased in the future climate scenarios as evidenced by the increase of NP ranging from 40% to 92% higher than the present (Figure 8, Tables S2 and S3). Concurrently, as the NP rose, the density of these patches also increased, determined by the PD growing by more than 41.88% in the future. Additionally, the size of these patches also expanded, leading to greater edge areas, which is evidenced by a 216% increase in the LPI and a 143% rise in TE. However, the LSI remained stable and indicated simpler shape geometry in the future scenario. Notably, the patches are now closer to one another due to the increase in both patch size and number, as reflected by a more than 4% increase in the AI, further indicating greater proximity between the patches (Figure 8, Tables S2 and S3).

On the contrary, the western range exhibited contrasting results from the eastern range in response to future climate changes. Specifically, the suitable patches in the western range showed a significant increase as indicated by a 571.87% rise in the NP compared to the present (Figure 8, Tables S2 and S3). This increase in patches led to a corresponding rise in the density of the patches, as witnessed by increasing PD ranging from 574% to 756% across the respective timeframes and SSP scenarios. However, despite the increase in the number of patches, the size of these patches notably decreased, as reflected by a 28% reduction in the LPI due to climatic shifts. Additionally, due to the growing patches and decreasing sizes, it led to the increase of edge areas by more than 12.11% in the future. Furthermore, these patches also show an increase in shape complexities as witnessed by the increasing LSI in the future. The fragmentation is further highlighted by a decrease of over 7.51% in the AI, suggesting that the patches are now more distantly spaced from each other. Overall, fragmentation is evident in the western range, as suitable patches become small and increasingly scattered, located farther apart in the future scenario compared to the present.

## 4 Discussion

In recent years, the species assessment using multifaceted approaches has emerged as a crucial strategy for addressing key conservation challenges worldwide (McMahon et al. 2011; Sharma et al. 2024). The integration of these multifaceted biological approaches into conservation science has played a pivotal role in mitigating extinction risks for numerous threatened species (Sharma et al. 2024; Weeks et al. 2011). However, the concerning lack of such scientific interventions for reptiles is notable, particularly given that reptiles account for nearly one‐third of all tetrapods, with approximately 21% of reptile species currently under threat globally (Cox et al. 2022). Hence, greater emphasis on scientific assessments is essential for reptiles to uncover their critical traits and inform effective conservation practices (Meiri et al. 2023). In this context, the present research aims to elucidate and reveal the complete mitogenome while assessing the habitat suitability of the threatened *H. thurjii* , thereby contributing valuable insights for targeted conservation actions.

### 4.1 Mitogenomic Perspective

The mitogenomic insight of the monotypic species *H. thurjii* exhibits strand symmetry, aligning with patterns observed in other Batagurinae taxa and the broader Testudines mitogenomes (Anderson et al. 1982; Kundu et al. 2020). The mitochondrial genomes play a crucial role in organismal systematics, especially regarding gene arrangement (Zardoya and Meyer 1998). Therefore, the structural organization of mitochondrial genes evaluated in this study for *H. thurjii* is crucial for understanding physiological processes, molecular pathways, life history traits, and the evolutionary forces shaping genomic architecture across Testudines species. It is evidenced that variations within mitochondrial genes, including PCGs, rRNAs, and CR, serve as crucial markers for understanding genetic diversity within geoemydids (Suzuki and Hikida 2011; Vamberger et al. 2014). The codon distribution across PCGs, along with the specific initiation and termination codons, reveals the nuances of protein synthesis and codon usage bias, which may influence gene expression efficiency (Kundu et al. 2019). Additionally, detailed investigations into nonsynonymous (Ka) and synonymous (Ks) substitution rates in these genes provide valuable insights into the species' evolutionary resilience, particularly its adaptive responses to fluctuating environmental pressures (Kundu, Kumar, Tyagi, et al. 2018). Hence, the current mitogenomic characterization of *H. thurjii* not only elucidates the evolutionary pathways of this endangered freshwater turtle but also underscores the intricate relationship between genetic mutations, selective forces, and their contributions to the evolution of PCGs.

Additionally, the CR of the mitochondrial genome is of particular importance due to its dynamic nature, exhibiting significant variability and a high adenine‐thymine (AT) content, thus a key area of interest in genomic studies. The repeat‐rich elements within the CR exhibit considerable variability and are characterized by specific motifs that are likely to form stable hairpin loops (Satoh et al. 2016). These loop structures are hypothesized to function as sequence‐specific signals involved in the termination of mitochondrial DNA replication. Various mechanisms influence the CR, such as gene rearrangements occurring through dual replication events, the formation of dimeric mitogenomes, and both random and non‐random gene losses. These processes play a crucial role in shaping the structural diversity of mitochondrial genomes and contribute to our understanding of the evolutionary mechanisms driving mitochondrial genome evolution (Bernacki and Kilpatrick 2020; Kundu et al. 2023). In this context, the observed AT‐rich bias, conserved motifs, and tandem repeats within the CR of *H. thurjii* are consistent with patterns seen in other Testudines species. This provides valuable insights into the functional and evolutionary aspects of mitochondrial genomics. The detailed characterization of the CR in *H. thurjii* , along with a comparative analysis of other species within the Batagurinae subfamily, will enhance our understanding of mitochondrial DNA replication and transcription regulation, population genetics, and evolutionary studies.

Furthermore, the evolutionary relationships, origins, and diversification of Testudines have garnered considerable attention in global research (Crawford et al. 2015; Shaffer et al. 2017). Previous studies have constructed a detailed phylogeny of all extant Testudines species, correlating their evolutionary diversity with historical climatic changes along Earth's continental margins (Thomson et al. 2021; Le et al. 2007). However, mitochondrial genomic data have proven invaluable in elucidating the evolutionary relationships of various Testudines species, including members of the Geoemydidae family and the Batagurinae subfamily (Feng et al. 2017; Kundu et al. 2019, 2020). The resulting phylogenies are consistent with earlier cladistic and evolutionary analyses, reaffirming the monophyletic clustering of the Batagurinae species within the broader geoemydid phylogeny. In particular, mitogenomic‐based cladistic analyses of the monotypic *H. thurjii* have revealed its divergence prior to the separation of *Batagur* congeners (Spinks et al. 2004; Praschag et al. 2007). Nevertheless, further mitogenomic data from the remaining Batagurinae species are necessary to refine and confirm the precise matrilineal relationships within this group of Testudines.

The comprehensive mitogenomic analysis of *H. thurjii* is essential for advancing species and population‐level identification, while also contributing to the development of informed conservation strategies (Kolbe et al. 2012; Harris et al. 2019). The current genetic findings provide a critical basis for future phylogeographic studies on *H. thurjii* , allowing for the comparison of nucleotide variations across various mitochondrial genes from diverse populations within its native range. This will be particularly valuable for the breeding of this highly endangered species, as it helps mitigate risks associated with inbreeding depression, the founder effect, and demographic stochasticity. Nevertheless, large‐scale population genetic data will further augment the understanding and management of potentially inbred populations of the endangered *H. thurjii* in India and adjacent regions. These initiatives will facilitate comprehensive assessments of the species' existing genetic diversity and provide valuable insights for scientific breeding programs and reintroduction efforts in South Asia.

### 4.2 Habitat Dynamics Perspective

Besides, the habitat suitability assessment identifies a mere 10.32% of the total IUCN‐designated extent as suitable for *H. thurjii* under current climatic conditions. Specifically, the western range constitutes a significantly larger suitable area (83,723 km^2^.) compared to the eastern range (35,757 km^2^.). However, it is crucial to emphasize that the delineated areas do not confirm the species' actual presence but rather highlight regions that share similar ecological and environmental conditions with the species' known niche. Furthermore, the model highlights the critical influence of bioclimatic variables, particularly Mean Temperature of the Driest Quarter (bio_9) and Annual Mean Temperature (bio_1), which contribute 19.51% and 16.64%, respectively, to the predictive model. These findings corroborate the established understanding that environmental parameters play a pivotal role in determining the distribution patterns of reptile species (Biber et al. 2023; Dayananda et al. 2021). Moreover, the assessment identifies proximity to waterbodies with a 5.10% contribution as one of the significant factors influencing habitat suitability for *H. thurjii* . This finding underscores the critical role of conserving riparian zones and adjacent riverine habitats to ensure the survival of this freshwater turtle species (Buhlmann et al. 2009). Intriguingly, the model indicates a substantial expansion in habitat suitability for the species under future climatic scenarios, with an overall increase of up to 118.32% across the designated extent. This expansion is predominantly concentrated in the eastern range, where suitability is projected to rise by over 183.64%. In contrast, the western range is anticipated to experience a decline in habitat suitability exceeding 27.27%. These shifts are driven by climatic changes, resulting in a redistribution of suitable habitats toward eastern Asia in the future (You et al. 2022). Additionally, the spatial geometry of suitable patches is predicted to undergo significant changes. In the eastern range, the increase in habitat suitability is characterized by larger patches with higher spatial proximity. Conversely, in the western range, the decline in suitable area is accompanied by increased fragmentation, with patches becoming smaller and more isolated. These findings highlight the necessity of directing conservation efforts toward regions exhibiting increased suitability as potential climatic refugia while addressing fragmentation in areas of decline (Durance and Ormerod 2007). Furthermore, the expansion in the eastern range emphasizes the importance of integrating habitat management with ongoing river conservation programs in the region (Hussain et al. 2020).

### 4.3 Recommendations for Conservation Implication

The present study provides a foundational baseline for mitogenomic and ecological data, facilitating species identification, population genetics, and conservation strategies for *H. thurjii* . However, as the current mitogenomic data for *H. thurjii* are derived exclusively from its eastern range, it is recommended that comparable genetic data be generated from its western range to achieve a more comprehensive population‐level understanding. Beyond mitogenomic insights, phylogenomic analyses incorporating nuclear genes and whole‐genome sequencing would yield a more robust understanding of the evolutionary history and genetic composition within the broader Testudines lineage. Additionally, identifying priority conservation regions under current and projected future climatic scenarios provides critical guidance for spatial conservation planning. These findings enable targeted conservation efforts in areas with the highest potential for supporting the species' long‐term persistence. While future climatic models suggest an expansion of suitable habitat, it remains imperative to mitigate anthropogenic pressures, including hunting and illegal trade, to ensure effective species conservation (Ahmed et al. 2021). Addressing these threats necessitates a multi‐stakeholder approach, involving collaboration among research institutions, conservation organizations, and wildlife trafficking control agencies across South Asia. This study further emphasizes the urgent need to protect riparian zones, which are increasingly vulnerable to anthropogenic activities such as silt and stone extraction, industrial waste disposal, etc. that pose significant threats to freshwater turtles. Furthermore, riverbank erosion, sedimentation, and various anthropogenic pressures are identified as key drivers of land use and land cover changes within the IGB River Basin, both presently and in the future (Cheema and Bastiaanssen 2010; Collins et al. 2013; Younis and Ammar 2018; Debnath et al. 2023). Therefore, all developmental activities within these ecologically sensitive regions, particularly the critical habitats along the Saryu River, should undergo comprehensive Environmental Impact Assessments (EIA) to assess and mitigate potential risks to freshwater turtle populations. Additionally, strict regulations should be enforced against harmful fishing practices, such as electrocution and the use of nylon nets, which have severe adverse effects on freshwater turtles and other aquatic fauna. Moreover, community engagement and awareness initiatives are essential to reducing destructive activities such as illegal turtle hunting and egg collection. Thus, implementing community‐based educational programs and outreach initiatives will be indispensable for promoting sustainable coexistence between human populations and freshwater turtles. These conservation measures will not only safeguard freshwater turtle populations but will also enhance the overall health and resilience of riverine ecosystems across South Asia by protecting these ecologically important scavengers.

## 5 Conclusions

The ongoing global freshwater crisis underscores the urgent need for the conservation of aquatic species, particularly ancient and highly threatened chelonians. This study presents the first complete mitogenomic analysis of the monotypic *H. thurjii* , offering novel insights into its genomic structure, sequence variation, and its matrilineal phylogenetic placement within the family Geoemydidae. These genetic findings contribute to understanding the evolutionary history of *H. thurjii* and closely related Batagurinae species. Furthermore, the integration of an ensemble‐based SDM approach provides a comprehensive perspective on the ecological dynamics of *H. thurjii* under current and projected future climate scenarios across its eastern and western ranges in Southern Asia. Collectively, this multidisciplinary approach provides a robust scientific foundation for the formulation of evidence‐based conservation and management strategies for *H. thurjii* and other freshwater turtle species globally.

## Author Contributions

**Imon Abedin:** conceptualization (equal), data curation (supporting), formal analysis (supporting), methodology (equal), software (supporting), writing – original draft (equal). **Arunima Singh:** data curation (supporting), methodology (equal). **Jayaditya Purakayastha:** formal analysis (supporting), software (supporting), writing – review and editing (supporting). **Shailendra Singh:** data curation (supporting), investigation (equal), validation (equal), writing – review and editing (supporting). **Kulendra Chandra Das:** investigation (equal), validation (equal), writing – review and editing (supporting). **Hyun‐Woo Kim:** funding acquisition (equal), project administration (equal), resources (equal), supervision (equal). **Hye‐Eun Kang:** funding acquisition (equal), software (supporting), visualization (equal). **Shantanu Kundu:** conceptualization (equal), formal analysis (supporting), project administration (equal), resources (equal), supervision (equal), visualization (equal), writing – original draft (equal), writing – review and editing (supporting).

## Conflicts of Interest

The authors declare no conflicts of interest.

## Supporting information


Data S1.

## Data Availability

The mitogenomic data used for the analysis were obtained from the open‐access GenBank database (Accession No. PP336441). The ecological modeling data are provided both in the main text and in the Supporting Information.
