# Supplementary material for: Unveiling the Evolutionary Lineages and Habitat Dynamics of the Monotypic Crowned River Turtle Hardella thurjii (Gray, 1831) (Testudines: Geoemydidae): Strategic Conservation Insights for an Endangered Freshwater Turtle From Southern Asia
Source: Ecol Evol. 2025 Jun 20;15(6):e71530. doi: 10.1002/ece3.71530 (PMC12179677; doi:10.1002/ece3.71530)
Supplement: Supplementary file 1 — Data S1. [file ECE3-15-e71530-s001.docx]

**SUPPORTING INFORMATION**


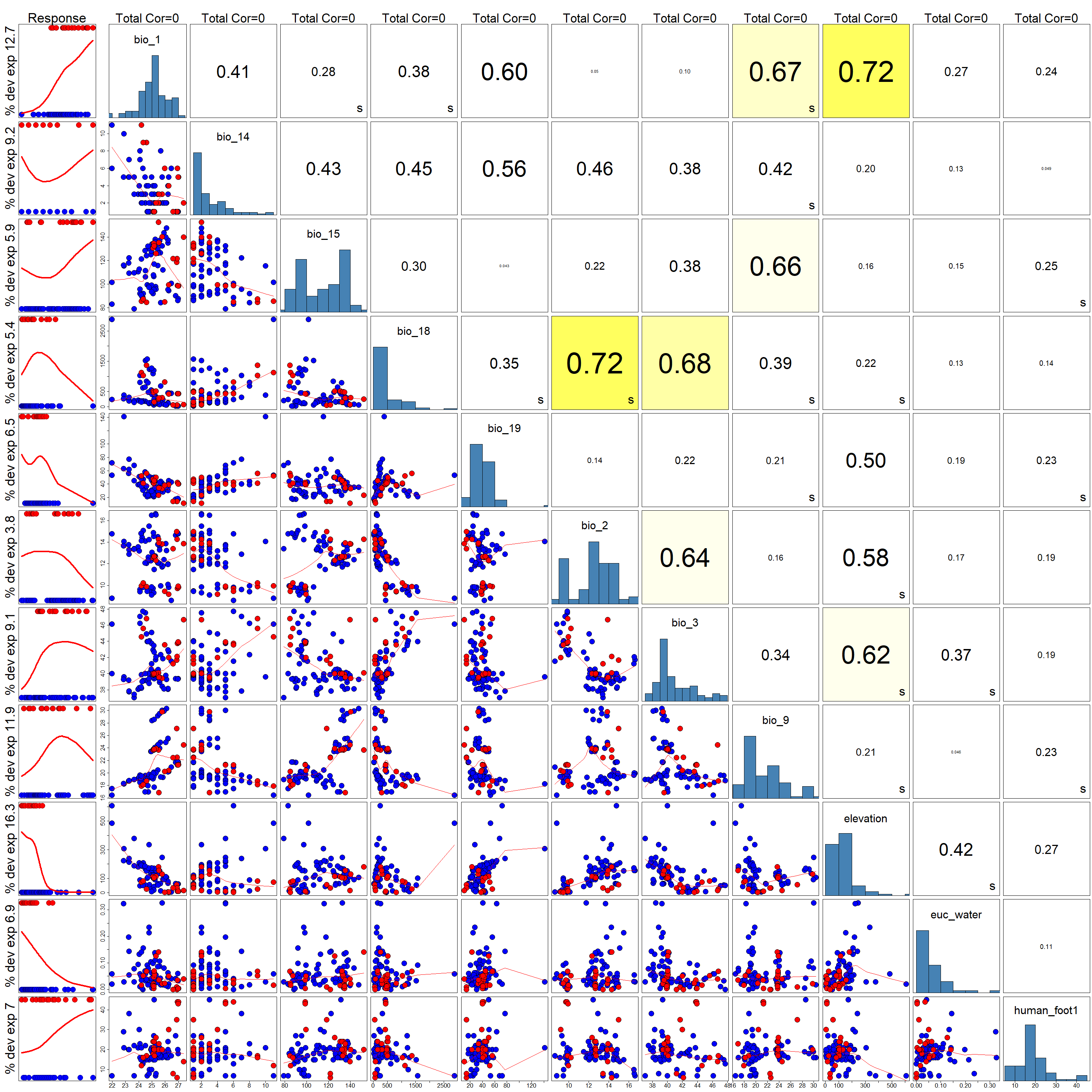


**Figure S1** Figure showing the correlation between the covariates chosen for final model for *H. thurjii*.


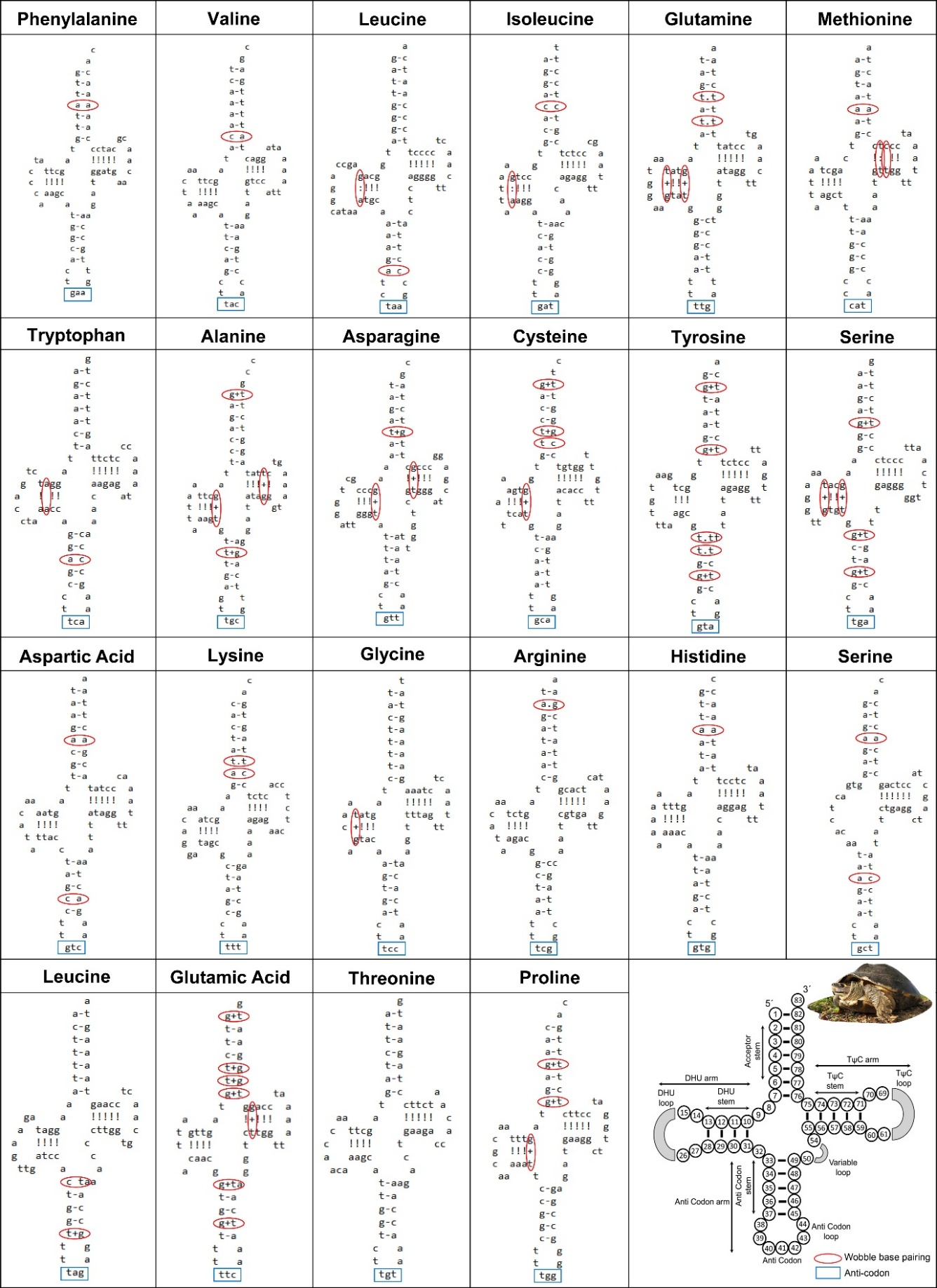


**Figure S2.** The secondary structures of 22 transfer RNA genes (tRNAs) of *H. thurjii* mitogenome display the structural variations. The last structure shows the nucleotide position and details of the tRNA stem-loop configuration. The wobble base pairs and anticodons are marked with red oval and blue rectangle, respectively.


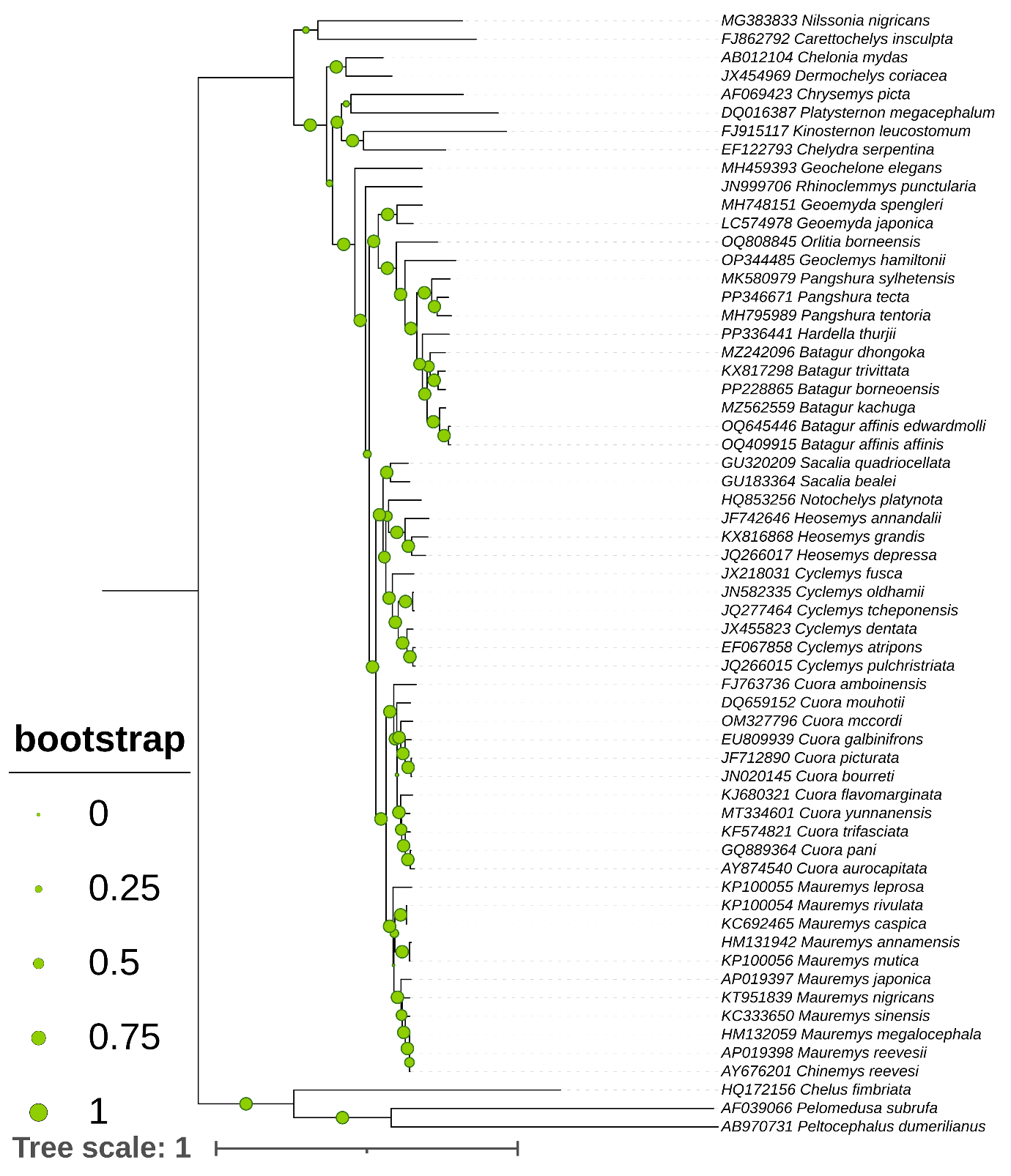


**Figure S3** The Maximum-Likelihood (ML) phylogenetic tree illustrates the major evolutionary relationships *H. thurjii* and other Testudines. Bootstrap supports values are represented by light green circular dots of varying sizes superimposed on each node.

**Table S1** Details of the mitogenome sequences generated and acquired from GenBank database for this study.

| **Sl. No.** | **Sub-Order** | **Family** | **Subfamily** | **Species Name** | **Accession No.** |
| --- | --- | --- | --- | --- | --- |
| 1 | Cryptodira | Geoemydidae | Batagurinae | *Hardella thurjii* | PP336441 |
| 2 | Cryptodira | Geoemydidae | Batagurinae | *Batagur affinis affinis* | OQ409915 |
| 3 | Cryptodira | Geoemydidae | Batagurinae | *Batagur affinis edwardmolli* | OQ645446 |
| 4 | Cryptodira | Geoemydidae | Batagurinae | *Batagur borneoensis* | PP228865 |
| 5 | Cryptodira | Geoemydidae | Batagurinae | *Batagur dhongoka* | MZ242096 |
| 6 | Cryptodira | Geoemydidae | Batagurinae | *Batagur kachuga* | MZ562559 |
| 7 | Cryptodira | Geoemydidae | Batagurinae | *Batagur trivittata* | KX817298 |
| 8 | Cryptodira | Geoemydidae | Batagurinae | *Geoclemys hamiltonii* | OP344485 |
| 9 | Cryptodira | Geoemydidae | Batagurinae | *Orlitia borneensis* | OQ808845 |
| 10 | Cryptodira | Geoemydidae | Batagurinae | *Pangshura sylhetensis* | MK580979 |
| 11 | Cryptodira | Geoemydidae | Batagurinae | *Pangshura tecta* | PP346671 |
| 12 | Cryptodira | Geoemydidae | Batagurinae | *Pangshura tentoria* | MH795989 |
| 13 | Cryptodira | Geoemydidae | Geoemydinae | *Cuora amboinensis* | FJ763736 |
| 14 | Cryptodira | Geoemydidae | Geoemydinae | *Cuora aurocapitata* | AY874540 |
| 15 | Cryptodira | Geoemydidae | Geoemydinae | *Cuora bourreti* | JN020145 |
| 16 | Cryptodira | Geoemydidae | Geoemydinae | *Cuora flavomarginata* | KJ680321 |
| 17 | Cryptodira | Geoemydidae | Geoemydinae | *Cuora galbinifrons* | EU809939 |
| 18 | Cryptodira | Geoemydidae | Geoemydinae | *Cuora mccordi* | OM327796 |
| 19 | Cryptodira | Geoemydidae | Geoemydinae | *Cuora mouhotii* | DQ659152 |
| 20 | Cryptodira | Geoemydidae | Geoemydinae | *Cuora pani* | GQ889364 |
| 21 | Cryptodira | Geoemydidae | Geoemydinae | *Cuora picturata* | JF712890 |
| 22 | Cryptodira | Geoemydidae | Geoemydinae | *Cuora trifasciata* | KF574821 |
| 23 | Cryptodira | Geoemydidae | Geoemydinae | *Cuora yunnanensis* | MT334601 |
| 24 | Cryptodira | Geoemydidae | Geoemydinae | *Cyclemys atripons* | EF067858 |
| 25 | Cryptodira | Geoemydidae | Geoemydinae | *Cyclemys dentata* | JX455823 |
| 26 | Cryptodira | Geoemydidae | Geoemydinae | *Cyclemys oldhamii* | JN582335 |
| 27 | Cryptodira | Geoemydidae | Geoemydinae | *Cyclemys pulchristriata* | JQ266015 |
| 28 | Cryptodira | Geoemydidae | Geoemydinae | *Cyclemys tcheponensis* | JQ277464 |
| 29 | Cryptodira | Geoemydidae | Geoemydinae | *Cyclemys fusca* | JX218031 |
| 30 | Cryptodira | Geoemydidae | Geoemydinae | *Geoemyda japonica* | LC574978 |
| 31 | Cryptodira | Geoemydidae | Geoemydinae | *Geoemyda spengleri* | MH748151 |
| 32 | Cryptodira | Geoemydidae | Geoemydinae | *Heosemys annandalii* | JF742646 |
| 33 | Cryptodira | Geoemydidae | Geoemydinae | *Heosemys depressa* | JQ266017 |
| 34 | Cryptodira | Geoemydidae | Geoemydinae | *Heosemys grandis* | KX816868 |
| 35 | Cryptodira | Geoemydidae | Geoemydinae | *Mauremys annamensis* | HM131942 |
| 36 | Cryptodira | Geoemydidae | Geoemydinae | *Mauremys caspica* | KC692465 |
| 37 | Cryptodira | Geoemydidae | Geoemydinae | *Mauremys japonica* | AP019397 |
| 38 | Cryptodira | Geoemydidae | Geoemydinae | *Mauremys leprosa* | KP100055 |
| 39 | Cryptodira | Geoemydidae | Geoemydinae | *Mauremys megalocephala* | HM132059 |
| 40 | Cryptodira | Geoemydidae | Geoemydinae | *Mauremys mutica* | KP100056 |
| 41 | Cryptodira | Geoemydidae | Geoemydinae | *Mauremys nigricans* | KT951839 |
| 42 | Cryptodira | Geoemydidae | Geoemydinae | *Mauremys reevesii* | AP019398 |
| 43 | Cryptodira | Geoemydidae | Geoemydinae | *Mauremys rivulata* | KP100054 |
| 44 | Cryptodira | Geoemydidae | Geoemydinae | *Mauremys sinensis* | KC333650 |
| 45 | Cryptodira | Geoemydidae | Geoemydinae | *Notochelys platynota* | HQ853256 |
| 46 | Cryptodira | Geoemydidae | Geoemydinae | *Sacalia bealei* | GU183364 |
| 47 | Cryptodira | Geoemydidae | Geoemydinae | *Sacalia quadriocellata* | GU320209 |
| 48 | Cryptodira | Geoemydidae | Rhinoclemmydinae | *Rhinoclemmys punctularia* | JN999706 |
| 49 | Cryptodira | Trionychidae | - | *Nilssonia nigricans* | MG383833 |
| 50 | Cryptodira | Carettochelyidae | - | *Carettochelys insculpta* | FJ862792 |
| 51 | Cryptodira | Kinosternidae | - | *Kinosternon leucostomum* | FJ915117 |
| 52 | Cryptodira | Chelydridae | - | *Chelydra serpentina* | EF122793 |
| 53 | Cryptodira | Dermochelyidae | - | *Dermochelys coriacea* | JX454969 |
| 54 | Cryptodira | Cheloniidae | - | *Chelonia mydas* | AB012104 |
| 55 | Cryptodira | Testudinidae | - | *Geochelone elegans* | MH459393 |
| 56 | Cryptodira | Platysternidae | - | *Platysternon megacephalum* | DQ016387 |
| 57 | Cryptodira | Emydidae | - | *Chrysemys picta* | AF069423 |
| 58 | Pleurodira | Podocnemididae | - | *Peltocephalus dumerilianus* | AB970731 |
| 59 | Pleurodira | Pelomedusidae | - | *Pelomedusa subrufa* | AF039066 |
| 60 | Pleurodira | Chelidae | - | *Chelus fimbriata* | HQ172156 |

**Table S2** The table represents the habitat quality and geometry of the suitable areas within the Eastern range of *H. thurjii* in present and future climatic scenarios. SSP: Shared Socioeconomic Pathways; NP: Number of patches; PD: Patch density; LPI: Largest patch index; TE: Total edge; LSI: Landscape shape index; AI: aggregate index.

| **Scenario** | **NP** | **PD** | **LPI** | **TE** | **LSI** | **AI** |
| --- | --- | --- | --- | --- | --- | --- |
| Present | 138 | 3285729 | 1.7594 | 46.6 | 14.7669 | 90.5754 |
| SSP 245 (2041-2060) | 266 | 6392382 | 5.5755 | 118.968 | 19.0345 | 94.3115 |
| SSP 245 (2061-2080) | 240 | 5767563 | 21.2611 | 122.28 | 16.3498 | 96.1923 |
| SSP 585 (2041-2060) | 194 | 4662113 | 11.5856 | 113.424 | 16.6883 | 95.6567 |
| SSP 585 (2061-2080) | 302 | 7257516 | 34.2676 | 144.368 | 14.4539 | 97.3531 |

**Table S3** The table represents the habitat quality and geometry of the suitable areas within the Western range of *H. thurjii* in present and future climatic scenarios. SSP: Shared Socioeconomic Pathways; NP: Number of patches; PD: Patch density; LPI: Largest patch index; TE: Total edge; LSI: Landscape shape index; AI: aggregate index.

| **Scenario** | **NP** | **PD** | **LPI** | **TE** | **LSI** | **AI** |
| --- | --- | --- | --- | --- | --- | --- |
| Present | 32 | 1207143 | 19.7448 | 30.96 | 6.5851 | 98.0488 |
| SSP 245 (2041-2060) | 239 | 9052767 | 13.2517 | 37.264 | 7.7715 | 90.1408 |
| SSP 245 (2061-2080) | 273 | 10340608 | 14.2144 | 40.488 | 8.2126 | 90.0623 |
| SSP 585 (2041-2060) | 230 | 8711868 | 12.1701 | 34.712 | 7.5108 | 90.1625 |
| SSP 585 (2061-2080) | 215 | 8143702 | 11.7451 | 41.152 | 8.457 | 90.6822 |
